# Cardiovascular disease as a biomarker for an increased risk of COVID-19 infection and related poor prognosis

**DOI:** 10.2217/bmm-2020-0201

**Published:** 2020-05-19

**Authors:** Nicola Ielapi, Noemi Licastro, Michele Provenzano, Michele Andreucci, Stefano de Franciscis, Raffaele Serra

**Affiliations:** ^1^Interuniversity Center of Phlebolymphology (CIFL). International Research & Educational Program in Clinical & Experimental Biotechnology at the Department of Medical & Surgical Sciences University Magna Graecia of Catanzaro, Viale Europa 88100 Catanzaro, Italy; ^2^Sapienza University of Rome, Department of Public Health & Infectious Disease, Roma, Italy; ^3^Department of Medical & Surgical Sciences University Magna Graecia of Catanzaro, Viale Europa 88100 Catanzaro, Italy; ^4^Department of Health Sciences University Magna Graecia of Catanzaro, Viale Europa 88100 Catanzaro, Italy

**Keywords:** 2019-nCov, cardiovascular disease, COVID-19, heart, infection, vascular

In December 2019, a novel coronavirus (2019-nCov) caused a pneumonia disease outbreak in Wuhan, China that became a pandemic. The 2019-nCov belongs to the coronavirus family and was classified in the beta-coronavirus 2b lineage. Coronaviruses are enveloped, nonsegmented positive-sense RNA viruses and are widely distributed in humans and other mammals. Typical symptoms include fever, nonproductive cough, dyspnea, fatigue and pneumonia [1–3], sharing several similar symptoms with severe acute respiratory syndrome, coronavirus and Middle East respiratory syndrome coronavirus, with all three diseases appearing to be zoonotic in origin [4,5]. While 2019-nCov frequently induces mild symptoms, physicians are particularly concerned about the severe symptoms and the elevated risk of death in coronavirus disease of 2019 (COVID-19) patients with cardiovascular disease, as these patients also seem to be more susceptible to this viral infection [6–8]. In this editorial we are going to discuss whether cardiovascular disease may be considered as a biomarker for an increased risk of COVID-19 infection and related poor prognosis.

## Cardiovascular involvement

Pre-existing cardiovascular comorbidities in COVID-19 patients, include hypertension (up to 40% of patients) [1,4,9,10], coronary heart disease (up to 10%), heart failure (up to 4%) and cardiac arrhythmias (up to 17%) [11–13]. Patients presenting more severe clinical manifestations demonstrated comorbidities such as hypertension (58%), heart disease (25%) and arrhythmia (44%) [1,8]. Overall, patients with cardiovascular disease represent more than 20% of all fatal cases, with a case fatality rate of 10.5% [12]. On the other hand, cardiovascular manifestations, during COVID-19, are mostly represented by acute cardiac injury (ACI), defined as a significant elevation of cardiac troponins in up to 12% of patients and arrhythmia in nearly 17% of patients. The potential long-term consequences on the cardiovascular system of patients who recover from this disease are not yet known, but the importance of the effect of COVID-19 infection on the cardiovascular system is also reflected through the elevation of high-sensitivity troponin I levels, novel ECG and echocardiogram abnormalities that can be evaluated during ACI [8,14,15].

COVID-19 patients are also at an increased risk of venous thromboembolism and there is evidence of alterations of the main coagulation parameters (elevated D-Dimer levels, fibrin degradation products), especially in patients with severe manifestations [16]. Furthermore, episodes of disseminated intravascular coagulation were also recorded [17].

## Pathophysiological considerations

2019-nCov has the ability to target cells by binding to angiotensin-converting enzyme 2 (ACE2); a membrane-bound amino-peptidase that is highly expressed in the cardiovascular system and can trigger direct myocardial injury. ACE2 is pivotal in physiologic neurohumoral regulation of the cardiovascular system and has an important role in cardiovascular disease. The binding of 2019-nCov to ACE2 may affect ACE2 signaling pathways, leading to ACI. Specifically, a patients susceptibility to 2019-nCov may depend on a higher expression of ACE2, that has been found in patients with hypertension and cardiovascular disease [15,16]. In fact, ACE2 can also be found in the media of diseased blood vessels as well as in angiogenic vessels, indicating a possible role in blood vessel remodeling and therefore this may be involved in atherogenesis and in other pathological vessel conditions [18]. In fact, elevated plasma ACE2 activity is an independent predictor of major cardiac events [19], correlating with cardiovascular disease development [20] and ACE2 was also found in carotid atherosclerosis and abdominal aortic aneurysm [21,22]

Another mechanism that could affect the cardiovascular system, as well as other bodily systems during the COVID-19 pandemic, is acute systemic inflammatory response caused by uncontrolled release of pro-inflammatory cytokines. Several studies have demonstrated the presence of a pro-inflammatory cytokines storm, particularly in patients with severe and critical manifestations, as IL-6, IL-10 and tumor necrosis factor-α (TNF-α) were found to be markedly higher in these patients. IL-6 alone was even elevated in moderate cases [23]. Moreover, systemic inflammation, as well as increased vascular shear stress at the level of coronary arteries can also trigger plaque rupture ad subsequent acute myocardial infarction [15].

Another mechanism that can sustain inflammatory based injury may be antibody dependent enhancement. Patients with a high inflammatory response may have been exposed for the very first time to one or a previous virus similar to coronavirus and because of antigenic epitope heterogeneity, the virus-specific antibodies, instead of being protective may enhance the entry of the virus and in some cases, even the replication of the virus [7]. Furthermore, electrolyte imbalances can also occur during the critical, systemic 2019-nCov infection and precipitate or induce cardiac arrhythmias [15].

Interestingly, during atherosclerosis related diseases, the functions and effects of ACE2 are also mediated by metalloproteinase (MP) families, such as matrix metalloproteinase (MMP), a disintegrin and metalloproteinase (ADAMs) families. In particular, MMP-2, MMP-3, MMP-9, and ADAM-17 have strict effects on ACE2 activity [14,24]. These MPs are also related to cytokine recruitment and therefore to inflammation, in particular IL-6 and TNF-α that are particularly active in the 2019-nCov infection [23].

## Expert commentary

The importance of this article lays on its capacity to stimulate researchers and physicians to deepen research into cardiovascular issues affected by COVID-19 and translating this to clinical practice. The 2019-nCov virus shares several common mechanisms with atherosclerotic diseases and their complications. For example, patients with cardiovascular disease, especially those related to atherosclerosis, offer the 2019-nCov virus a fertile ground to initiate and progress infection of the host. In this context, cardiovascular patients represent the most fragile part of the population, as these patients are more susceptible to 2019-nCov infection and its dreadful complications. ACE2 related mechanism, MP control systems and cytokine levels are often dysregulated in cardiovascular disease [15,16,23]. Moreover, mechanisms related to ACE regulations and inflammation are complex and closely related [23,24].

The more comprised the vascular system becomes, the more likely the 2019-nCov infection will have devastating effects. For example, COVID-19 patients with acute coronary syndrome will certainly have a poor prognosis due to morphologic and hemodynamic damage to heart tissues. In these cases, cardiac insufficiency may occur rapidly, leading to a sudden deterioration and fatal complications. For these patients COVID-19, can act as a precipitating factor to worsen their condition, and lead to fatality [14].

Furthermore, considering clinical and therapeutic implications, several concerns were raised among physicians and patients regarding the intake of inhibitors of the renin–angiotensin–aldosterone system in COVID-19 patients, as these drugs can upregulate ACE2. However, it is not clear enough at this time whether ACE2 expression necessarily correlates with the severity of 2019-nCov infection. Therefore, at the moment, it is not advisable to stop renin–angiotensin–aldosterone system intake as, it may even increase the overall risk of cardiovascular mortality [25]. For venous thromboembolism, anticoagulant treatment with low molecular weight heparin is also associated with decreased mortality, especially in severe clinical manifestations of the 2019-nCov infection [26].

While we are quite sure about the clinical relationship between the heart and 2019-nCov infection, we need to perform further investigations to determine the clinical implications of other diseases related to the vascular system such as, aneurysm, peripheral artery disease and carotid stenosis. Patients with the aforementioned comorbidities may experience a higher susceptibility to infection and to related complications, as well as deterioration of the vascular disease (for example, aneurysm rupture, carotid plaque vulnerability with subsequent stroke and acute limb ischemia), as the same inflammation biomarkers are also present in these conditions [27,28].
